# How to Study Religion? Notes on Research Methodology in the Context of Latin American Religions

**DOI:** 10.1007/s41603-022-00188-0

**Published:** 2023-01-23

**Authors:** Donizete Rodrigues

**Affiliations:** Center for Research in Anthropology, Nova University of Lisbon, Lisbon, Portugal

**Keywords:** Methodology, Ethnography, Fieldwork, Netnography, Latin American religions

## Abstract

The aim of this paper is to discuss on the main methodological procedures used in Anthropology and Sociology and applied in studies of Latin American religions, particularly in the context of diasporic Brazilian Protestantism-Pentecostalism. After introduce the two principal categories (quantitative and qualitative) – which include various types of procedures, such as the case study, interdisciplinary, historical, comparative and cross-cultural—and based on ethnographic experiences of the author in America, Europe and Asia, examine the world scale and ‘glocal’ multi-situated ethnography and the traditional localized participant-observation, including the ‘outsider-insider dichotomy’ and Asia, *the article* examines. However, today, with new digital technologies and the broad cultural and religious manifestations in the Internet, the researcher can complement the search for information (ethnographic data)—and accompany of the daily life of the group, of the community under study—using the Internet, the various social networks, namely, Facebook, Twitter, and WhatsApp. The principal contribution of this work is to present the specificities of the ethnographic field in the studies of religious movements, evangelical churches, in particular, where issues and problems posed to researchers requiring appropriate ethical and methodological procedures for overcoming them.

## Introduction

Firstly, it is relevant to explain that this text is not an exhaustive discussion about research methodologies in the study of religion—many other authors have already discussed this issue in a more complete and detailed way (Dillon 2003; Davie 2007; Stausberg and Engler 2011)—but only bring some notes on the main methodological procedures used in Anthropology and Sociology and applied in studies of Latin American religions, particularly in the context of diasporic Brazilian Protestant-Pentecostalism.

After introduce the two principal categories (quantitative and qualitative) – which include various types of procedures, such as the case study, interdisciplinary, historical, comparative and cross-cultural—and based on ethnographic experiences of the author in America, Europe and Asia, examine the world scale and ‘glocal’ multi-situated ethnography and the traditional localized participant-observation, including the ‘outsider-insider dichotomy’. However, today, with new digital technologies and the broad cultural and religious manifestations in the Internet, the researcher can complement the search for information (ethnographic data)—and accompany of the daily life of the group of the community under study—using the Internet and the various social networks, namely, Facebook, Twitter, and WhatsApp.

As I mentioned above, the methodological procedures discuss here are related to the studies of religion, which is “one of the most important social forces in any society” (Day 2020:vii). Trying to respond the pertinent question what does it mean and why to study religion in the present time (Ellwood 2016), in a very broad sense, and based on classic and contemporary anthropological and sociological perspectives (Harding and Rodrigues 2014), I consider that the studies of religion are very important for the understanding of the complex relationship between religion and society, particularly how the religious people organize their daily life, construct their worldview, and tempt to influence and make social and religious changes (for better world, according to them) in their groups/communities and the society in general.

In this specific case, there are three principal theoretical-methodological procedures (Brink 1995; Thorton 2022: (a) phenomenological (the Weberian substantive theory), it concerns of essence, the meaning and nature of religion, and the experiential aspect of the religious phenomenon; (b) the study of religious groups and communities (the Durkheimian functionalism theory); and (c) lived religion/a *religion vécue*, with a holistic perspective, i.e., the study of all aspects of the beliefs, practices, and daily spiritual experiences of the followers of a particular religion movement.

## The First Stage: Design the Project

Although there is not a unique or ideal methodological arrangement, a reasonable approach to a problem in social sciences is the following (Bechhofer and Paterson 2000; Hunt and Colander 2017):

1. Define the problem and objectives of the project.
2. Review the literature, the “state of the art” to become familiar with what others already have studied.
3. Develop a theoretical framework based on classical and contemporary authors and theories.
4. Choose the research design—methodologies and techniques.
5. Collect the data—empirical fieldwork.
6. Analyze the results.
7. Draw conclusions and write the final report (paper/article, dissertation, thesis, book).

In the design of a scientific research project, the choice of appropriate methodology is determined by the proposed objectives: what kind of information the researcher wants or needs to obtain and, paraphrasing Bauman (2000), writing sociologically on?

## The Different Types of Methods

The method is a body of practices, procedures, and rules used in the process of searching data and information, in a specific knowledge field. In the case of the social sciences (Anthropology and Sociology), based on theories and previous empirical observations, social scientists use the method as an integrated strategy to organize their research practices (Bechhofer and Paterson 2000).

The method helps in the selection, observation, collection, classification, and analysis of social phenomena, that is, of social reality (Riis 2009). The techniques, in turn, are the instruments and practices for collecting and processing information, and they include oral, documents, film/video, photography, and statistical data.

Quantitative and qualitative constitute the predominant and the most relevant methodological categories; however, there are other important methods that we can use in the social sciences, as the following (Hunt and Colander 2017).

*Case study*: involves making a detailed study and analysis of a specific issue or problem situation. Case studies can be qualitative, quantitative, or be both together.

*The interdisciplinary approach*: a group of researchers from different fields of study work together on a particular issue or problem.

*The historical method*: tracing the principal past events that seem to have been directly significant in bringing about a present social-cultural-religious situation. This is the concept that the knowledge of the past can give insights into present circumstances and even to future trends.

*Comparative and cross-cultural methods*: the comparison of different societies, groups, and phenomenon play an important role in anthropological and sociological studies. The method consists of making detailed research of the social/cultural/religious and behavioral patterns of the members of societies for the purpose of comparing the similarities among them.

Let me get back to the discussion of *quantitative* and *qualitative* methods, my focus in this part of the paper.

Specifically, in social sciences, there are two principal and well-known methods, i.e., two interrelated modes of producing knowledge: quantitative and qualitative. Although they can be associated (“quali-quanti” combined procedures)—according to Riis (2012), divide between quantitative and qualitative methods is unclear, unnecessary, and unfruitful—generally speaking, the quantitative is considered to be more sociological (macro-social analysis), while the qualitative is more anthropological (micro-social perspective).

Quantitative studies use surveys, polls, and questionnaires aiming to obtain data and information. It involves social characteristics as variables (subject to standardized measurement and attributed with numeric values), statistical analysis of data in numeral form that has been gathered and classified, which provide material capable to understand the broad social and cultural processes and even some particular meanings.

A pertinent question here is: why quantitative data are appropriate and important to the study of religion? Because it provides the quantification of trends and patterns of religious life and collective behavior (Stausberg and Engler 2011), moreover, according to Brink (1995), in general, quantitative data have the advantage of giving relatively precise hypothesis testing and are also less susceptible to the possibility of various interpretations.

Even considering that I do not use the quantitative data—math was never my favorite subject—in my research studies on religion, I use only the qualitative method. However, this personal option, from a methodological point of view, is not a problem: as I said above, the choice of methodology is determined by the previously defined objectives; indeed, the most important aspect is the type of information the researcher wants or needs to obtain.

Speaking now about the qualitative method, this procedure is useful if the main purpose is to follow a development closely over a period of time, and usually, it is geared to the study of specific social, cultural, and religious movements and relatively small groups and communities (Furseth and Repstad 2006; Riis 2009). Focused in two key questions “how” and “why,” the approaches provide a lot of rich descriptive detail that enables us to explore the meanings of belief and religious practice.

To conclude this part, it is important to retain two ideas: none methodological procedures is better than the other, and the researchers are not limited to only one, “because there is nothing especially sacred about either quantitative or qualitative methodologies” (Slife and Melling 2012, p. 732).

Considering that this text focus on particularly the Anthropology, let me first discuss its traditional qualitative method.

## Participant-Observation

In the empirical work, the most common qualitative technique used in Anthropology is the ethnography, i.e., the “anthropologist in the field” describing the groups/communities/societies and gathering information (Mauss 1947; Seligman 1951). Ethnography—a micro-sociology, from Weber and Simmel’s perspectives—is particularly interested in the relations between social actors, individuals or groups, and the positions and social roles that the actors occupy and play within the social milieu in which they are inserted.

In its beginning, to understand primitive societies, social/cultural Anthropology has developed a fieldwork procedure called “participant-observation,” a well-known term coined by Bronislaw Malinowski (1922), a Polish-born and English-based academic.

In the field, attempt to understand the viewpoint of a native culture (Clifford and Marcus 1986), the researcher does not merely observe, but also tries to integrate and fully participate in the daily activities of the group or society under study. This method involves prolonged observation, participation in all (or at least the principal) activities, and interviewing members of the group (DeWalt and DeWalt 2010; Bernard and Gravlee 2015). To be more specific, the.

fieldwork … describes the activities that take place in a particular research locale over the medium to long term … the researcher then studies the locale by living or working there for a period of time, or by making repeated visits (Bechhofer and Paterson 2000, p. 91).

As Evans-Pritchard (1937), doing ethnographic fieldwork in Central Africa with the Azande, testified:

I tried to adapt myself to their culture by living the life of my hosts, as far as convenient, and by sharing their hopes and joys, apathy and sorrows. In many respects my life was like theirs: I suffered their illnesses; exploited the same food supplies; and adopted as far as possible their own patterns of behavior with resultant enmities as well friendships (p. 45).

In the specific case of the study of religion, of the *homo religious*, Sociology and Anthropology, using the comparative method, study the great diversity of beliefs and religious practices in different social-cultural context. This approach always involves the symbols, myths, rites, rituals and experiences of the sacred lived in the context of society (Geertz 1973; Bowie 2000; Stausberg and Engler 2011; Stein and Stein 2017). When we are researching on a specific case of a religious movement, an evangelical church, for instance, this anthropological method allows us an empathic understanding, acting, worshiping, feeling with, and for living within the congregation (Rodrigues 2014).

However, in the context of participant observation, there are two categories, or better explaining, two principal ways for the researcher to immerse in the study group: “outsider” or “insider.”

### The “localized Study”: Outsider-Insider

In “localized studies,” the researchers—if they have conditions for it—can choose to be “outsider” or “insider.” Actually, the insider/outsider issue has been a subject for academic studies of religion since the beginnings of Sociology and Anthropology in the mid-nineteenth century (Arweck and Stringer 2002). For this reason, in the sociological and anthropological studies of religious movements, there is a large amount of literature discussing the importance insider–outsider dichotomies (Merton 1972; Geertz 1983; Headland et al. 1990; McCutcheon 1999; Ganiel and Mitchell 2006).

It is important to note that, methodologically, sometimes it is difficult to determine when the researcher is an outsider or insider, because there are different levels of “insiderness” and “outsiderness” (Arweck and Stringer 2002). Moreover, many sociologists and anthropologists argue the relationship of the terms *insider* and *outsider* as being less of a dichotomy and more as a continuum.

An important methodological question to consider is advantages bestowed upon and disadvantages besetting a researcher, as an insider, or as an outsider. While the outsider is excluded from many (and probably the most important) religious activities and discourse, the insider is enabled and equipped, to more fully, and from a first-hand point of view, to understand the religious group (“the tribe”) and their spiritual experiences (Ganiel and Mitchell 2006). As an insider, it is easier for the researcher to be accepted as one of them. As Geertz (1983) emphasizes in his interpretative anthropology, studying religion (as a system of symbols) in inextricably linked to its socio-cultural milieu, the outsider can *observe*, but only the insider can *feel* the human behavior and the religious experience (p. 60).

In the case of the study of religious movements (e.g., evangelical churches), this methodological strategy is extremely important: it allows active and prolonged participation in numerous social and religious events, special cults, prayer groups, vigils, and spiritual retreats. It is pertinent to point out that many of these activities (spiritual retreats, in particular) are only allowed to members that are effective, active, more dedicated, and committed to the church and to a faith community. In a study that I conducted on a Brazilian evangelical church in the New York metropolitan area, not only I used the observation-participant, but I was also “insider-believer ethnographer” (Rodrigues 2014).

The specific fieldwork methodology used in Anthropology—the “participant-observation,” being “outsider” or “insider” ethnographer—remains today the prevalent means of research in the “localized situations.” However, in the present times, there is a new paradigm, called ‘multi-sited ethnography’, a term coined by George Marcus (1995). He argues that the study of social/cultural matters cannot be adequately accounted for by focusing on a single site/place, like the traditional old studies on indigenous village. Let me explain better what multi-site ethnography is.

## Multi-Site Ethnography

When the study is about migrations of people and religious groups in diasporic context, exemplified in this writing with the case of the global expansion of Brazilian religious movements (Rocha and Vasquez. 2013; Rodrigues and Oro 2014; Oosterbaan et al. 2019), which necessary involves a geographic dimension of world scale: what is the most appropriate methodology to study a phenomenon of such magnitude? For certain, the answer is the “multi-sited ethnography,” because it is most appropriate in the study of the grand scale of migrations (people and religions) with transcontinental dimensions.

According to Falzon (2009), “The essence of multi-sited research is to follow people, connections, associations, and relationships across space … multi-sited ethnography involves a spatially dispersed field through which the ethnographer moves” (p. 1–2). From the same perspective of “macro ethnography” (Appadurai 1996), it is a kind of “traveling anthropology.” In this case, the ethnographer makes multi-sited fieldwork, mapping routes, pathways, and flows rather than describing fixed and localize cultures.

In the context of post-modernity, replacing the (old) location (or located place) by multiple and dispersed places, the multi-sited ethnography focuses on translocal phenomena, diasporic situations, such as transnational migration flows, including people and religious movements. Much more than places, borders, and localized situations, the emphasis is the spatial mobility and the multiple locations and activities. The important objective is to follow the trajectories of a specific phenomenon, making connections and links between the elements, for example, the relationship between immigration and religion (Coleman and Hellerman 2011). In this context, while following people and religious movements, the researcher also becomes a traveler or, more specifically, a “traveler-ethnographer” (Appadurai 1996).

In a first phase, the researcher is a traveling ethnographer, mapping and meeting immigrants, and agencies and religious groups, in various geographic contexts. After this macro-ethnographic approach, as a second phase, the research prioritizes, in the respective countries, the localized situations, doing fieldwork with the religious groups/churches. As I mentioned earlier above, in the localized situations, by using the traditional participant observation, there is another methodological option: the ethnographer can be an outsider, insider, or use both practices/research perspectives.

There is another important issue that we can highlight here: how can site-specific participant observation and multi-sited ethnography approaches could be combined?

The multi-sited ethnography and participant observation are two different methodological procedures. Yet the researcher can easily compound them, without losing the specificity of each measure. Speaking about my experiences, in my studies on Brazilian evangelical movements’ expansion to USA, Europe, and Japan, I have been used both approaches. Let me explain better:

- In the first step, I do multi-sited/macro-ethnography research, as a “traveler-ethnographer,” following the transnational flow of the religious movement. In this first prospective ethnography, the primary source of information is the specialized mission training agencies for pastors to evangelize in other countries. Another source is the official sites of the religious movements, which bring data and detailed information on their worldwide expansion.
- In some cases, I do fieldwork in “localized situations,” using the method of participant-observation, being “outsider” or, in some specifically circumstances, a “native-insider-believer.” The meaning that I obtain from emic research, as an insider-believer, is much more relevant than of “mere observation,” because it grants me a fully participation in all activities of the churches and congregations (Rodrigues 2014).

In my experiences, I have had many situations as follows: what role the researcher should have in the fieldwork, and what responsibilities and tasks have been assigned to the ethnographer by leaders, pastors, and believers in the church structure and routine? After the followers (membership) consider that the “outsider” is already “converted,” what is the place the researcher should occupy in the church/congregation? To be more specific, what are their expectations about the new “born again,” in particular in the important task of converting others? Due to the complexity of these inquiries, combined with the specificities of the various types of religious movement, these kinds of questions are not easily adequately answered. However, as I mentioned above, the explanation depends on the procedure adopted: whether the researcher is being “outsider” or “insider” makes a big epistemological and methodological differences.

The methodological procedures discussed above basically refer to qualitative research, geographically located and with long-term engagement and physical presence of the researcher in the field (Thorton 2022). However, localized ethnography, in the classical perspective of Malinowski (1922) and, later, Geertz (1973), is predominant, but not the only one possible.

### The Use of the Internet in the Study of Religion

According to Clifford Geertz’s interpretive Anthropology, ethnography is not merely a research methodology or a simply data collection technique. In fact, ethnography has a broader approach, focusing on the understanding of societies and groups, based on a “thick description” of the sociocultural and religious practices. The ethnographer, thus, aims to explain how these practices, experiences, and social dynamics constitute “webs of significance” (Geertz 1973).

Ethnographic fieldwork, in the traditional perspective of Malinowski and, later, of Geertz, implied (compelled) the long-term physical presence of the anthropologist in the field. However, today, with new digital technologies, the researcher can complement the search for information (ethnographic data)—and accompany of the daily life of the group of the community under study—using the Internet, the various social networks, namely, Facebook, Twitter, YouTube, and WhatsApp.

In the 1990s, to answer the need to adapt the classical ethnographic research method (in/of the geographic terrain) to the study of the virtual world (in/of digital media) and its methodological implications, several concepts and approaches emerged: netnography, virtual ethnography, digital ethnography, online ethnography, webnography, and cyberanthropology (Hjorth et al 2019).

In this context, a prominent term is netnography, being Robert Kozinets (2015) the greatest exponent. This author considers netnography (“internet ethnography” or “online ethnography”) as a specific type of qualitative research on social media/networks, which adapts the methodological procedures used in participant observation to the study of subcultures, groups and virtual communities, and the social interaction that occurs in the computer‐mediated communication. These interactions and experiences, which are manifested through digital communications, constitute the primary source of data and information for the ethnographic understanding of a particular social, cultural (and religious) phenomenon.

It is pertinent to emphasize that the first studies on social interactions on the Internet had an ethnographic base. Christine Hine, with the work virtual ethnography (2000), was one of the pioneers. According to Hine, cyberspace can be studied as culture, in the anthropological perspective. According to this view, the Internet is also a place where culture is (re)constituted. Thus, it is the role of the ethnographer, as an insider in the research context, in direct contact with the interlocutors, to study the phenomena and cultural practices that take place in the virtual communities.

With regard specifically to the application of online ethnography in the studies of religion, after the great development and massive use of the Internet on a worldwide scale, churches, historical religions, and thousands of new religious movements (NRMs) began to use this important means of communication (mass media) to hold and share religious events and spread their religious messages with proselytize purpose and to convert people. There are even churches and NRMs that only exist on the Internet, without occupying a physical, real place—they are the so-called “virtual churches,” “digital religion,” “religion online,” and “online religion” (Campbell and Tsuria 2021).

In cyberspace, people can, in a de-territorialized and timeless way, read about religion; interview religious leaders and followers; talk to other people about religion and their mystical-religious experiences (chat, conversation group); download sacred books (e.g., the Bible) and religious documents; view images and videos; listen to religious music, sermons, and testimonies; practice meditation; participate in virtual pilgrimages and religious rituals; and attend religious ceremonies, masses, and worship services (Hadden and Cowan 2000; Dawson and Cowan 2004; Hojsgaard and Warburg 2005).

At the end of 2019, a global biological occurrence altered radically the social life of all societies in the world. A dangerous virus has completely altered people’s lives, isolating them in cities, in neighborhoods, and at home. The serious pandemic situation caused by the coronavirus-19 has profoundly transformed people’s daily lives, causing significant changes in behavior, including the way religious communities engage in worship services. As Day (2020) pointed out, “the world was shuddering with the horrific impact of a pandemic. Lives, livehoods, faiths and certainties where shattered” (viii). Huygens (2021) adds: “in the times of COVID-19, it is no longer possible to invoke bodily engagements such as attending church services, going on a pilgrimage, experiencing sensory stimulations such as smelling incense, listening to Church hymns, or tasting the wafer” (p. 6).

As I mentioned above, the intensive use of Internet (social media) for religious reasons, especially by Christian churches and groups, to proselytization, hold, and share religious activities, was very common before the pandemic crisis. The implementation of lockdowns and strict social distancing protocols pressed strongly the religious movements and their leaders to invest even more in livestream platform to broadcast worship services online (Campbell 2020; Isetti 2022).

Yet, this new situation brought new challenges, especially to the Pentecostal movements, but also to afro-Brazilian religions that only recently begun to occupy the social media platform (Capponi and Araújo 2020; Lima 2022): considering the impossibility to attend physically places of worship and religious practices, how to deal with the important issue of the embodied participation in worship? As Campbell (2020) highlighted, using worship online, how to accomplish, the “Eucharist,” a special spiritual moment of the assembled congregation, which involves embodied elements of the incarnation? Isetti (2022) adds, would it be possible a kind of “cyber-Eucharist”? (p.2). There are authors, such as Hutching (2011) and Addo (2021), among others, who believe that is possible some form of “digital eucharist” (or “eucharist online”), with more relevance in Pentecostal communities. Nevertheless, Isetti (2022) argues that “in digital religion studies one of the main concerns that is raised” is “whether ‘disembodied’ or mediated interactions online can ever truly allow for an authentic religious expression and experience online” (p.2). As Huygens (2021) testified, “despite all these (online and remote) alternatives to physical gatherings, all my interlocutors stated that ‘it is not the same’” (p.6).

Quarantine and social distancing, caused by the pandemic, brought an enormous challenge to religious movements, namely, those working in diasporic contexts. However, their leaders, imaginative and with a great capacity for innovation and adaptation, easily migrated from physical to online spaces, finding thus new ways to express their religious devotion (Campbell 2020). As Addo (2021) imaginatively expressed, “the believers become digital spiritual hype people” (p. 53).

To close this idea, with regard specifically to religious practices, from the emergence of the pandemic crisis, religious groups, who are already very well-trained and experienced in practicing religion online, began to use even more the digital spaces to continue to be in contact with the followers, give spiritual support to them, and to take the religious message to a wider audience.

At the present time, in the called “post-COVID era,” with large vaccination protocols, the social distancing measures are being lifted. As a consequence, the faithful are returning to their “old” physical temples; however, negotiating the boundaries between online and offline spaces, they do not abdicate the very attractive and efficient “digital altar” (Addo 2021).

Another consequence of pandemic situation is that the academic people were (physically) separated from their primary work place—the university (where they teach) and from the ethnographic terrain (where they do research). From the methodological point of view, with the impossibility of real, physical contact, what would be the alternative for ethnographic research? The possible choice is to use social networks to accompany the daily lives of the followers, groups, and religious communities.

To conclude this brief methodological approach on the use of the internet in the study of religion, it is important to remember that Anthropology, throughout its history, has managed to adapt, epistemologically and methodologically, to historical and new cultural changes. The social impact of COVID-19, this invisible and deadly enemy for humanity has brought a new methodological challenge, but also new possibilities for ethnographic research. In a context of an increasingly digital world, ethnography had to adapt effectively, flexibly, and creatively to this new social and religious realities. And the specialized bibliography proves that this necessary methodological turn is being very successful.

When the researchers are in the field, physical and/or virtual, studying religious movements emerged many questions, doubts, and indecisions. And they observe also contradictions and have tensions moments and ethical dilemmas (Hale 2001). They often are bothered and even shocked by some “weird” or strange situations; in fact, each one of us has a story to report. Without intending to exhaust the situations, I discuss now some issues and problems that usually emerge in (my) fieldwork.^1^

## The Ethnographic Field: Methodological Issues and Problems

In order to discuss methodological issues and research problem in the ethnographic field—and the dissemination of the results—I considered several sociological and anthropological studies, classic and contemporary ones (see the bibliography). From this critical reading—and also based on my fieldworks experience—I formulated the following methodological procedures (Rodrigues 2018). With these practices, I intend to help researchers who are begin their social and cultural studies, on religion, in particular.

1. In the initial formulation and execution of a project, the researchers should go to the ethnographic work with a set of initial questions. However, they must be able to adapt them according to the reality on the field. In fact, at the end of the process, they leave the field and return to the office even with more questions/inquiries and uncertainties. As Thorton (2022) pointed out, “starting a new project required me to be flexible, adapt my approach, and alter my initial expectations” (p. 23).
2. When the ethnographer arrives for the first time in the field, it is necessary to carefully choose two important interlocutors, who will define the relative success (or lack thereof) of the study: the *gatekeeper* and *key-informants*. Speaking about gatekeeper first: “this is a data collection term and refers to the individual who the researcher must visit before entering a group or cultural site. To gain access, the researcher must receive this individual’s approval.” That is, the initial contact is made in negotiation with gatekeepers who retain the decision and the power of access to the “tribe” to the church and religious community/group. In the case of key-informants, “they are individuals with whom the researcher begins in data collection because they are well informed, accessible, and can provide leads about other information” (Creswell 1998, p. 247).
3. One outstanding experience of participant observation in a different cultural context is the so-called cultural shock.^2^ It implies the ethnographer’s difficulty in adapting to a new culture/society/religion/group context: receiving and transmitting wrong behavioral signals; not being able to have an appropriate conduct; and dealing with behaviors of the Other, which are—in the view of the ethnographer’s original culture—inappropriate, shocking, dirty, immoral, or simply different, but which are perfectly normal and acceptable within the social-cultural-religious context in which he/she is temporarily inserted.
4. It is common in the fieldwork that the ethnographers have been considered as socially and culturally misfit by the society that they are studying. With another process of socialization/endoculturation, and are unaware of the basic rules of sociability of the group under study, they have to learn the social rules of the new community where they are now inserted for some period of time.
5. Diaries and field notes are the primary techniques of recording data collected in the fieldwork. As Malinowski (1922) put it, “to bring home to the reader all the conditions in which … the observations were made” (p. 3). In addition, the researcher should take photographs, record (in audio and video) informal conversations and events, conduct interviews, and observe behaviors. These practices are primordial for recording of everyday cultural-religious practices and posterior construction of anthropological narrative and analysis of the group under study (Geertz 1988).
6. The ethnographer’s personal characteristics—such as ethnic/racial, nationality, identity, gender, age, social class, sexual orientation, religious affiliation—have a considerable (positive or negative) impact on fieldwork. I am Portuguese-Brazilian, and this attribute makes it easier for me to be accepted by Brazilian religious movements in the diaspora, evangelical churches, in particular. In this case, my ethnic-identity-linguistic characteristic greatly facilitated my insertion in the different evangelical congregations that I have been studied; I always was presented and seen by the followers and pastors as “Brazilian-evangelical-immigrant in need of religious-spiritual support” (field notes).

But look at another situation: in my study, in Portugal, of the Church of Philadelphia, a Neo-Pentecostal congregation, an ethnic-church whose membership are almost 100% gypsy families, the fact that I am not ethnically gypsy made me unable to better immersion in the community, that is, being an “insider”; thus, the methodological alternative was to be outsider. In this case, did not sharing the same ethnic affiliation, I stayed outside of the community, which restricted the access to valuable data, information, and some rituals.

Ethnic affiliation raises another question: the language. Do the researchers speak the language of the evangelical “tribe” that they are studying? For better immersion in the religious group/community under study, it is very important to be able to dialogue, without translators/intermediaries, with the followers; only that way the researcher can more easily gain the confidence of the key-informants and be able to share personal matters, collect testimonies, and participate in religious experiences.

In my studies on Brazilian immigrants, in the diasporic context in the USA, Europe, and Japan, and more recently on the expansion of evangelical churches among indigenous peoples in Amazon region (Rodrigues and Moraes 2018), the question of language is not a problem. However, the situation changed dramatically when I started to study, in 2015, the Tenrikyo, a Japanese Shinto movement, and a Pentecostal church in Cambodia, in 2016. In both situations, not speaking the native language, the alternative was to use translators/interlocutors. In the case of the New Life Church, in Phnom Penh, the founding pastor himself, of American origin, even living in the country for several years, does not speak Khmer; to overcome the situation, he nominated a native pastor to lead the cults and do the work of proselytism and evangelization. Therefore, being an “ethnic church,” religious ceremonies and worship are celebrated in the native language. For this reason, my study on this church was more sociological than anthropological; the preliminary ethnography was made in English and more observation than participation.

7. Although it may be uncomfortable to talk about personal matters, it is important that the researchers, when questioned directly by the “natives,” answer sincerely to questions about their religious beliefs/practices, opinions, and values. However, they should share opinions and personal information in the most neutral way as possible; it means never trying to influence the natives.
8. Taking into consideration the Weberian and Durkheimian methodological orientation, it is important to note that the study requires that the researchers abandon all their social, cultural, and religious prejudices in order not to influence the scientific explanations. The fieldwork, closely associated with the collection and interpretation of cultural practices (Geertz 1983, 1988), challenges us to reflect critically on an important epistemological issue: the ethnographic look and reading could be filtered, distorted, or colored by our own ideas and feelings about religious orientation and world view.

In this context, studying religion, there is an imperative measure: the ethnographer must constantly be alert and self-conscious about ethical neutrality regarding the claim of “true/false.” That is, we must be “interested in what people *say* is the truth, the way people *think* the world works, their *understanding* of the mysteries of God … and their actual *behaviour*” (Bowie 2000, p. 49). In a very respectful manner, our concern should never be to test the “truth”: of belief, of religious practices, of ritual efficacy, or of judging divergent biblical interpretations (Wilson 1982). That is, we do not need to agree with, or to approve, in order to seek to understand the religious practices and meaning.

It is pertinent to remember here the Weberian epistemological/methodological procedure called “axiological neutrality” (Wertfreiheit). Axiological neutrality means that scientific research is exclusively valid for intrinsic reasons, by virtue of methods and procedures that are based on its specificity in the demonstration and verification of the scientific proposals. Despite the difficulty (or even impossibility) of a pure objectivity, the researchers, in any circumstances, should never make value judgments and condemn or approve the social and cultural practices (religion in particular) that they are studying.

9. Reciprocity is another important element of fieldwork. It involves the ethnographers to foster the livelihood and well-being to help the “natives” in many things, such as government-bureaucratic matters, to share personal property, information, and service provision, transporting people, if they are able to. Reciprocity also includes sharing the final results of the research with the community/group. This is essential for participatory research (action and applied Anthropology) and is especially important if the community studied can make good use of the sociological and anthropological information to solve some of their daily problems. For example, when I was doing my PhD thesis in social anthropology at Coimbra University, I spent 3 years (1988–1990) in a small peasant village in the interior of Portugal. Following the logic of reciprocity, I helped the locals with bureaucratic matters, shared information and data (the history of the village, land tenure, the birth/marriage/death registers, kinship relations, etc.), and transport in my car people to medical treatment and visit healers/witches very far from the village.

It is pertinent to refer here another situation of reciprocity. Because of my studies on gypsy communities in Portugal and Romania, between 2000 and 2007 (Rodrigues 2006b), I participated in several academic meetings and conferences in Portugal, Hungary, and Cuba, promoted and sponsored by the European Parliament, and I contributed also to create European laws and regulations to establish the rights of Roma as an ethnic minority, including freedom of religious expression.

10. Because of the enormous circulation of information and knowledge dissemination, on a world scale, a pertinent question in this reality is: how much of a concern could it be for the researchers when the interlocutors (particularly, the pastors and leaders) read what was written about the religious group/church? Indeed, this issue is not new. Anthropologists have long faced a challenge that affects how they produce and disseminate the results of their study and their knowledge: “natives” can read the final text and may or may not agree with the interpretations/explanations given by them. Today, more and more people are connected to the Internet and consult information (texts, photographs, videos) available online. Considering that many of the scientific-academic publications are now accessible through electronic sources and devices, the work of the anthropologists can easily be previously and/or subsequently scrutinized by the individuals and groups involved in their research.

Yet there are two specific circumstances here: before or after the publication. Let me present example of both situations:

- I once had a situation *before the publication*: the pastor-leader (the gatekeeper) of the First Baptist Church (localized at Marília, São Paulo, Brazil) placed a condition before allowing the research; he wanted to follow closely and read the final text with the results/explanations of the research, and we concurred (Rodrigues and Mendes 2018). The leader of the church only authorized the publication after making some minor changes, pointing out some “inaccuracies” in our explanations, but not enough to substantially modify the anthropological analysis. In this specific case, the result was very positive; because reading the preliminary text, he made several clarifications and brought new questions, contributing thus to a better anthropological interpretation of the object of study.
- The case of *after publication* is more common: The Universal Church of the Kingdom of God, a religious, economic, and media empire, a Brazilian religious movement that I have been studying for two decades (Rodrigues 2006a), is highly professional, especially in its relationship with media, social communication, and also academic publications. They have a specialized department that accompanies registers, catalog, and everything that is published about the church. The fact is that the leaders want to know how and what the journalist and academic are opining and writing about them and the UCKG.
- Another important issue in ethnographic research is the ethical procedure of confidentiality. In the identification of the group, place, and people mentioned in the study, the researcher-author should use fictitious names, that is, should “anonymize” them in order to protect their identities.
- There is a diverse set of scientific criteria to evaluate whether the empirical work was well done or not: the method, techniques, and concepts were well applied. The researcher was able to properly narrate the situation, event, and ceremonies. The description was sufficiently rich and detailed. The description followed objective criteria, and it is consistent with other data collected on the field. The locals, native, and believers agreed with the results and the interpretations. There are others explanations and interpretations that could be considered for the same cultural, social phenomenon, and religious practice. There are interpretations/explanations that can be generalized. The results created new theories/explanatory models and new methodological procedure.

In addition to these 12 methodological procedures, particularly applied in the study of religion, another pertinent question to bring here is: are there particular challenges in doing anthropology of religion that differ from what we can say, “activist anthropology”?

In my research on religious movements, and following the Weberian “wertfreiheit” that I discussed above, I try not to be an activist nor an applied anthropologist. Yet, it is important to highlight that although there are many similarities, “activist anthropology” and “applied anthropology” have not the same perspective.

In the applied Anthropology, anthropologists use their skill and knowledge to solve day-to-day native practical problems; “walking together while researching” is the well-known procedure. It provides a number of effective action strategies that can be used to assist community/groups in reaching goals and resolving their problems (Willingen 2002).

In the activist/engaged Anthropology, the researcher is strongly committed to the “tribe” problems. More than to walk alongside the people/communities and give them the anthropological skills to use for resolving their problems (Hale 2001; Low and Merry 2010), the anthropologists must actively defend—including politically, the interests of the natives in their relationship with the dominant society/group, for example, physical survival, demarcation of the lands, traditional health practices, and preservation of their cultural and religious identities. In the case of religious movements, it is usually the defense of their proselytism, religious practices, and public manifestations.

Trying to conclude this discussion on methodological procedure on religious studies, let me bring one more (and the final) issue: it is not easy to develop studies on religious movements without undergoing a sort of attempt at conversion, indoctrination, and expectations of proselytism; that is, during the time that the researcher is inserted in the group, doing the fieldwork, he/she suffers constant questioning and attempt of conversion. In my fieldwork on evangelical churches, I have heard many times, “when will you be baptized, becoming truly one of us?” (field notes).

## Final Considerations

The goal of this paper was to discuss on the main methodological approaches used in Anthropology and Sociology and applied in studies of Latin American religions, particularly the relationship between immigration (diaspora) and evangelical churches, theme that I have been doing ethnographic fieldwork in USA, many countries in the South Europe, and more recently in Asia (Japan and Cambodia).

I emphasized the two principal and general categories of methods, the quantitative and qualitative, which include various types of procedures, such as the case study, interdisciplinary, historical, comparative, and cross-cultural. Specifically, after the classical localized participant-observation, including the “outsider-insider dichotomy,” emerged a new paradigm, the world scale and “glocal” multi-situated ethnography, which is also used by Sociology and Geography. These traditional ethnographic proceeds implied (compelled) the physical presence of the anthropologist in the field. However, today, with new digital technologies and the broad cultural and religious manifestations in the Internet, intensified with the impact of the worldwide coronavirus pandemic, the researcher can complement the search for information (ethnographic data)—and accompany of the daily life of the group, of the community under study—using the Internet and the various social networks, namely, Zoom, Facebook, Twitter, and WhatsApp.

According to the vast bibliography, the religious studies involve a lot of personal difficulties and dilemmas (Blanes 2006). Considering that it was not an exhaustive approach, but only some notes on methodology and religion, in my perspective, the principal contribution of this work is to discuss the specificities of the ethnographic field in the context of studies of religious movements, evangelical churches in particular, where issues and problems are posed to researchers, requiring appropriate ethical and methodological procedures for overcoming them: negotiation with gatekeeper, choose the key-informants, characteristics and personal matter, socio-cultural-religious prejudices, axiological neutrality, reciprocity, writing, publication and scrutinization by the native people, activist/applied perspectives, attempts of conversion/indoctrination of the ethnographer, and so on.

It is pertinent to mention that I did not pretend to exhaust the discussion on production of scientific knowledge, nor the diversified strategies of research in Sociology and especially in Anthropology. In fact, considering the complexity of the theme, it is (almost) impossible to exhaust the subject in such a brief work. Based on my field experience, I only wanted to demonstrate the importance of discussing, in the context of empirical production, the necessary epistemological and methodological procedures that must be considered for a correct procedure in scientific work on religious studies.

From a theoretical approach and the formulation/presentation of some important methodological procedures that must be followed in the fieldwork, I intended in this text discuss how the research trajectory is processed, emphasizing some difficulties and problems that the researcher may face during the ethnography and possible solutions. Through this analysis and sharing my experiences, I hope to help young researchers, anthropologists, and sociologists in their difficult and complex task in the study of religion.

## Footnotes

1 Many of these issues and reflections came up in the fruitful academic debate, held in many doctoral seminars that I taught: All Souls College, Oxford University, England (2001); Department of Religion and Content and Methods of the Social Sciences Seminar, Columbia University, New York/USA (2009–2010, 2016); and Department of Sociology and Anthropology, Ben-Gurion University of the Negev, Israel (2018). My sincere thanks to Bryan Wilson†, Peter Clarke†, Courtney Bender, Sidney Greenfield, and Jackie Feldman; their constant encouragement, suggestions, and contributions made this work theoretical and methodologically much better. However, they are not responsible for probable inaccuracies and errors presents in this text.

2 It is pertinent to emphasize that this rule was more common at the beginning of Anthropology, in the first fieldwork of the early twentieth century, where the ethnographer’s “cultural shock” in contact with the native was more evident. To exemplify, I would like to mention here two situations: the Englishman Radcliffe-Brown, among the Andamanese (India) and Australian aboriginal people and the Polish Malinowski, among the Trobriand islanders of Papua New Guinea.
